# Thank you to Virology Journal's peer reviewers in 2013

**DOI:** 10.1186/1743-422X-11-4

**Published:** 2014-01-22

**Authors:** Linfa Wang

**Affiliations:** 1 CSIRO Animal Food and Health Sciences, Australian Animal Health Laboratory, Geelong, VIC, 3220, Australia; 2 Program in Emerging Infectious Diseases, Duke-NUS Graduate Medical School, Singapore, 169857, Singapore

## Abstract

The editors of *Virology Journal* would like to thank all our reviewers who have contributed to the journal in Volume 10 (2013). The success of any scientific journal depends on an effective and strict peer review process and *Virology Journal* could not operate without your contribution. We are grateful to the large number of reviewers (1026 to be exact!), who have done a great job in not only lifting the quality of the journal’s scientific peer reviewing process, but also helped us to achieve our goal of a median time to first decision of just 35 days.

Our record time from submission to online, open access, publication in 2013 was 22 days for a Research Article [1] and 28 days for a Review [2]. This is a great achievement by any standard. We look forward to your continuous support of *Virology Journal* either as an invited reviewer or a contributing author in the years to come.

## Contributing reviewers

Stephen Abedon

USA

Dharam Ablashi

USA

Jônatas Abrahão

Brazil

Sazaly AbuBakar

Malaysia

Ishag Adam

Sudan

Catherine Adamson

UK

Claudio Afonso

USA

Alexey Agranovsky

Russia

Jose Aguilar-Reina

Spain

Kamruddin Ahmed

Japan

Gillian Air

USA

Mitsuru Akashi

Japan

Gottfried Alber

Germany

Lorraine Albritton

USA

Alí Alejo

Spain

Amauri Alfieri

Brazil

David Allen

UK

Adelaide Almeida

Portugal

Gabriel Almeida

Brazil

Saeed Amel Jamedar

Iran

Abbe Ames

USA

Maria Joao Amorim

Portugal

Nisalak Ananda

Thailand

Matthew Angel

USA

Peter Angeletti

USA

Ana Angulo

Spain

Christophe Antoniewski

France

Miguel Aranda

Spain

Katherine Arden

Australia

Jiro Arikawa

Japan

Luciana Arruda

Brazil

Sassan Asgari

Australia

Usman ali Ashfaq

Pakistan

Houssam Attoui

UK

Prasert Auewarkul

Thailand

Kevin Ault

USA

Maysa Azzeh

Palestinian Territory

Eduardo Azziz-Baumgartner

USA

Shawn Babiuk

Canada

Leen Baert

Switzerland

Andre Bafica

Brazil

Olfa Bahri

Tunisia

Dalan Bailey

UK

Jean-Luc Bailly

France

Michelle Baker

Australia

Anne Balkema-Buschmann

Germany

Ashley Banyard

UK

Xiaoyong Bao

USA

Eric Baranowski

France

John Barnes

USA

John Barr

UK

Kelli Barr

USA

Sarah Barreto

Brazil

Alan Barrett

USA

Naina Barretto

USA

John Bashiruddin

UK

Chris Basler

USA

Armanda Bastos

South Africa

Daniel Bausch

USA

Lorena Bavia

Brazil

Maria Serena Beato

Italy

Jin-Xin Bei

China

Mark Beilke

USA

Sarah Belisle

USA

Brendan Bell

Canada

Jessica Belser

USA

Abdelouaheb Bennani

Morocco

Kirsten Bentley

UK

Sven M. Bergmann

Germany

Ben Berkhout

Netherlands

Jonathan Bertin

Canada

Jonathan Bertin

Canada

Walter Betancourt

Venezuela

Jayaram Bettadapura

Australia

Daniela Bezemer

Netherlands

Marcia Bianchi santos

Brazil

Eric Blair

UK

Esther Blanco

Spain

Gary Blissard

USA

Evan Bloch

USA

Sandra Blome

Germany

Thomas Bock

Germany

Barbara Boine

Germany

Myrna Bonaldo

Brazil

Andre Boonstra

Netherlands

Durlav Prasad Bora

India

Alessandra Borges

Brazil

Vanni Borghi

Italy

Akos Boros

Hungary

Berend Jan Bosch

Netherlands

Thomas Briese

USA

Christopher Broder

USA

Corrie Brown

USA

Dennis Brown

USA

Meredith Brown

USA

Glenn Browning

Australia

Robert Brueggeman

USA

Ivan Brukner

Canada

Shilpa Buch

USA

Chris Buck

USA

Marie Annick Buendia

France

Alexander Bukreyev

USA

Alexander Bukreyev

USA

Rowena Bull

Australia

Johan Burger

South Africa

Graham Burgess

Australia

Alison Burman

UK

Aurelia Busca

Canada

Marc Busch

USA

Sarah Butcher

Finland

Mathias Büttner

Germany

Charles Calisher

USA

Amanda Calvert

USA

Jennifer Cameron

USA

Grant Campbell

USA

Thomas Campbell

USA

Octavio Campollo

Mexico

Rodolfo Hector Campos

Argentina

Elena Canelli

Italy

Shengbo Cao

China

Alejandra Capozzo

Argentina

Margareth Capurro

Brazil

Antonella Caputo

Italy

Susan Carpenter

USA

Jill Carr

Australia

Ana Rita Castro

Brazil

Tatiana Castro

Brazil

Jamie Cate

UK

Mattia Cecchinato

Italy

Pieter-Jan Ceyssens

Belgium

Paul Chan

Hong Kong

Tak Mao Chan

Hong Kong

Yoke-Fun Chan

Malaysia

Jinhong Chang

USA

Juliana Chávez

Brazil

Isabelle Chemin

France

En-Qiang Chen

China

Feng Chen

USA

Jian Chen

USA

Pei-Jer Chen

Taiwan

Wuguo Chen

USA

Xinwen Chen

China

Ze Chen

China

Zhe-Sheng Chen

USA

Ju-Chien Cheng

Taiwan

V Gregory Chinchar

USA

Kyoung-Oh Cho

Korea, South

Ashok Chockalingam

USA

Il-Ryong Choi

Philippines

Kyoung-Seong Choi

Korea, South

Yeon Choi

USA

Young Ki Choi

Korea, South

Neil David Christensen

USA

Justin Jang Hann Chu

Singapore

Wan-Long Chuang

Taiwan

Massimo Ciccozzi

Italy

Luka Cicin-Sain

Germany

Jesse Clark

USA

Alan Cochrane

Canada

Aidan Coffey

Ireland

Manola Comar

Italy

Samuel Cordey

Switzerland

Marion Cornelissen

Netherlands

Adriana Correa

Brazil

Mathias Corteel

Belgium

Maria Isabel Costafreda

USA

Robert Coutts

UK

Lucyna Cova

France

Megan Crane

Australia

Lunbiao Cui

China

Claus-Peter Czerny

Germany

Flavio Da Fonseca

Brazil

Krystyna Dabrowska

Poland

Armando Damiani

Germany

Ying Dang

USA

Srikanta Dash

USA

Sibnarayan Datta

India

Alan Davis

USA

Andrew Davison

UK

Cecilia Dayaraj

India

Wu De

China

Cristian De Battisti

Italy

Luana de Borba

Argentina

Juan Carlos de la Torre

USA

Nicola Decaro

Italy

Sehgal Deepak

India

Aldo Dekker

Netherlands

Mariana del Vas

Argentina

Serena Delbue

Italy

Fei Deng

China

Yi-Mo Deng

Australia

Carl Deom

USA

Erik Depla

Belgium

Klaus Depner

Germany

Moutaz Derbala

Qatar

Daniel Desmecht

Belgium

Ulrich Desselberger

UK

Joanne Devlin

Australia

Francesco Di Serio

Italy

Marcel Dinger

Australia

Maurizio Divizia

Italy

Gerhard Dobler

Germany

Virginie Doceul

France

Riccardo Dolcetti

Italy

Joseph Domachowske

USA

Samuel Dominguez

USA

Àngela Domínguez

Spain

Chunsheng Dong

China

Anton Dormer

USA

Xiaoguang Dou

China

Joseph Dougherty

USA

Trevor Drew

UK

Jan Felix Drexler

Germany

William Drobyski

USA

Ioannis Drositis

Greece

Heidi Drummer

Australia

Edward Dubovi

USA

Jean-Bernard Duchemin

Australia

Robert Duda

USA

Jaquelin Dudley

USA

Joseph Dudley

USA

William Dundon

Austria

John Dunn

USA

Ralf Durrwald

Germany

Robert Dusek

USA

James Earnest

USA

Marion East

Germany

Andrew Easton

UK

Hideki Ebihara

USA

Diako Ebrahimi

Australia

Marcela Echavarria

Argentina

Iriane Eger

Brazil

Christina Ehrhardt

Germany

Awatef El Moussi

Tunisia

Gabriella Elia

Italy

Manal El-Sayed

Egypt

Christabel Enweronu-Laryea

Ghana

Koray Ergunay

Turkey

Michael Eschbaumer

Germany

Jose M Escribano

Spain

Susanna Esposito

Italy

Sandra Essbauer

Germany

Rosa Esteban

Spain

Magnus Evander

Sweden

Catherine Ewen

USA

Caio Fagundes

Ireland

DeLisa Fairweather

USA

Ann Falsey

USA

Shufang Fan

USA

Weihuan Fang

China

Mark Fast

Canada

Alberto Fereres

Spain

Brian Ferguson

UK

Humberto Fernandes

UK

Mark Fernandes

Singapore

Luca Ferrari

Italy

Jaqueline Maria Siqueira Ferreira

Brazil

Raina Fichorova

USA

Jonathan Filee

France

Cristina Fink

Brazil

Russell L. Finley Jr.

USA

Matthias Fischer

Germany

Uwe Fischer

Germany

Vincent Fondong

USA

Samuel Forster

Australia

Caroline Fossum

Sweden

Sandra Frabasile

Uruguay

Lori Frappier

USA

Roy French

USA

Zhen Fu

USA

Marc Fuchs

USA

Walter Fuchs

Germany

Masashi Fukayama

Japan

James Fung

Hong Kong

Alice Fusaro

Italy

Mohammed Gadour

Sudan

Carl A. Gagnon

Canada

Nicolas Gaidet

France

Ratish Gambhira

USA

Yulong Gao

China

Lorena Garaicoechea

Argentina

Urtzi Garaigorta

USA

Pilar García

Spain

Ana L. Garcia-Perez

Spain

Antonio Garmendia

USA

Carolina Garrido

USA

Aura Garrison

USA

Wolfgang Garten

Germany

Silvana Gaudieri

Australia

Riccardo Gavioli

Italy

Adam Geballe

USA

Brian Geiss

USA

Patrick Gérardin

Reunion

Jaquelline Germano de Oliveira

Brazil

Wilhelm Gerner

Austria

Maria Magdalena Gherardi

Argentina

Souvik Ghosh

Japan

Kathrin Gibbert

Germany

Robert Gilbertson

USA

Jason Gill

USA

Gennadi Glinsky

USA

Ricardo Gomez

Argentina

Yilei Gong

USA

Francisco Gonzalez-Scarano

USA

Steve Goodbourn

UK

Laura Goodman

USA

Felicia Goodrum

USA

Jennifer Gordon

USA

Elena Govorkova

USA

Sagar Goyal

USA

Beatrice Grasland

France

Christian Grund

Germany

Xingnian Gu

Australia

Rodrigo Guabiraba

UK

Yongjun Guan

USA

Jerome Guechot

France

Maria Isabel Guedes

Brazil

Gilles Guillemin

Australia

Susana Guix

Spain

Fang Guo

USA

Haitao Guo

USA

Xin Guo

China

Brandon Guthrie

USA

Bernd Haas

Germany

Roy Hall

Australia

Gunnel Hallden

UK

Saeed Hamid

Pakistan

Steve Hanson

USA

Chanditha Hapuarachchi

Sri Lanka

Timm Harder

Germany

Heli Harvala

UK

Hideki Hasegawa

Japan

Tayebeh Hashempoor

Iran

Daisuke Hayasaka

Japan

You-Wen He

USA

Klaus Hedman

Finland

Regine Heilbronn

Germany

Mark Heise

USA

Els Henckaerts

UK

Sander Herfst

Netherlands

Paul L Hermonat

USA

Michael Hess

Austria

Roger Hewson

UK

Eberhard Hildt

Germany

Ken Hirasawa

Canada

Wenzhe Ho

USA

Long Hoang

Singapore

Bernd Hoffmann

Germany

Per Hollsberg

Denmark

Hak Hotta

Japan

Weihong Hou

USA

Wendy Howard

UK

Eike-Roman Hrincius

Germany

John Hu

USA

John Hu

USA

Fen Huang

China

Liping Huang

China

Li-Rung Huang

Taiwan

Wenlin Huang

China

Yaowei Huang

USA

Austin Hughes

USA

Joseph Hughes

UK

Stephen Hughes

USA

Veijo Hukkanen

Finland

Abrar Hussain

Pakistan

Snawar Hussain

USA

Hala Ibrahem

Egypt

Hiroshi Ichimura

Japan

Ali Idris

USA

Enrique Iglesias

Cuba

Tetsuro Ikegami

USA

Tomozumi Imamichi

USA

M. Irshad

India

Pavel Isa

Mexico

Stuart Isaacs

USA

Miren Iturriza

UK

James Jancovich

USA

Christine Jansen

Netherlands

Hongyu Jia

China

Renyong Jia

China

Ping Jiang

China

Dong-Yan Jin

Hong Kong

Hui Jin

China

Emma Job

Australia

Reimar Johne

Germany

Clinton Jones

USA

Kathryn Jones

USA

Morris Jones

USA

Prapan Jutavijittum

Thailand

Tal Kafri

USA

Rolf Kaiser

Germany

Adriana Kajon

USA

Biao Kan

China

Sang-Moo Kang

USA

June Kan-Mitchell

USA

Michael Kann

France

Jia-Horng Kao

Taiwan

Vladimir Kapitonov

USA

Gerardo Kaplan

USA

Beatrix Kapusinszky

USA

Axel Karger

Germany

Hiroaki Kariwa

Japan

Fatah Kashanchi

USA

Benedikt Kaufer

Germany

Marcus Kehrli

USA

Guenther Keil

Germany

Alyson Kelvin

Canada

Lawrence Kenyon

Taiwan

Daniel Kern

USA

Pattara Khamrin

Thailand

Mohsin Khan

USA

Sunil Khattar

USA

Alan Khoo

Malaysia

Yury Khudyakov

USA

Frederick Kibenge

Canada

Tara Kieffer

USA

Byounghan Kim

Korea, South

Seong-Jun Kim

USA

Shin-Hee Kim

USA

Su-Mi Kim

Korea, South

Young Bong Kim

Korea, South

Brian Kimble

USA

Istvan Kiss

Sweden

Pravina Kitikoon

USA

PJ Klasse

USA

Aloysius Klingelhutz

USA

Nick Knowles

UK

Norbert Koch

Germany

Jeffrey Koehler

USA

Tuckweng Kok

Australia

Tee Kok Keng

Malaysia

Kouacou Konan

USA

Jeroen Kortekaas

Netherlands

George Koutsoudakis

Spain

Anna Kramvis

South Africa

Philip Krause

USA

Joost Kreijtz

Netherlands

Jan Kreuze

Peru

Susanne Kreuzer

Germany

Neel Krishna

USA

Bernd Kronenberger

Germany

Andrew Kropinski

Canada

Detlev H Kruger

Germany

Mart Krupovic

France

Chih-Hung Ku

Taiwan

Ondrej Kucera

Czech Republic

Beate Kuemmerer

Germany

Jens H. Kuhn

USA

Richard Kuhn

USA

Thijs Kuiken

Netherlands

Pankaj Kumar

USA

Sachin Kumar

India

Santosh Kumar

USA

Vinod Kumar

India

Kattareeya Kumthip

Thailand

Elizabeth Kutter

USA

Ann Kwan

Australia

Nicole La Gruta

Australia

Michael Lagunoff

USA

Ching Lung Lai

China

Amreek Lal

Pakistan

Elisabeth Lampe

Brazil

Daryl Lamson

USA

Jason LanmanJasonL

USA

Moshe Lapidot

Israel

Lars Larsen

Denmark

Magdalena Larska

Poland

Karen Laurie

Australia

Jen Layden

USA

Tiziana Lazzarotto

Italy

Ghislaine Le Gall-Recule

France

Jason LeBlanc

Canada

Neil LeBlanc

Sweden

Chang Lee

USA

Wai-ming Lee

USA

Porntippa Lekcharoensuk

Thailand

Guy Lemay

Canada

David Lembo

Italy

Keith Leppard

UK

George Leser

USA

Feng Li

China

Guoqing Li

China

Jisu Li

USA

Linlin Li

USA

Shifang Li

China

Xiangdong Li

China

Zejun Li

China

Zhenghe Li

China

Pawel P. Liberski

Poland

Daniel Libraty

USA

Uwe G. Liebert

Germany

Gipsi Lima Mendez

Belgium

Gongzhen Liu

China

Helene Minyi Liu

Taiwan

Hongqi Liu

China

Qinfang Liu

USA

Shuwen Liu

China

Wei Liu

China

Xiao-qing Liu

China

Yuanyuan Liu

USA

Zheng-Fei Liu

China

Francisco Lobo

Brazil

Thomas London

USA

Xavier Lopez

Spain

Marcelo Lopez-Lastra

Chile

Ben Lopman

UK

Carl Lowenberger

Canada

Zhengchun Lu

USA

Fabio Luciani

Australia

Min-Hua Luo

China

Guanggang Ma

China

Nabg Ma

Canada

Darin Madson

USA

Ken Maeda

Japan

Diogo Magnani

USA

Kathy Magor

Canada

Ravi Mahalingam

USA

Kristiina Mäkinen

Finland

Shinji Makino

USA

Rajinder Mann

USA

Fabricio Marchini

Brazil

Philip Marcus

USA

Francois Maree

South Africa

Vazeille Marie

France

Joao Marques

Brazil

Anthony Marriott

UK

Glenn Marsh

Australia

Emily Martin

USA

Isidoro Martinez

Spain

Miguel Angel Martinez

Spain

Encarna Martinez Salas

Spain

Luis Martinez-Sobrido

USA

Rodrigo Martino

Spain

Masaji Mase

Japan

William Mason

USA

Yumiko Matsuoka

USA

David Matthews

UK

Jelle Matthijnssens

Belgium

Leena Maunula

Finland

Damien Maura

USA

Wendy Maury

USA

Jens Mayer

Germany

Julie McAuley

Australia

John McCauley

UK

Myra McClure

UK

Christopher McCormick

UK

Jonathan McCullers

USA

Duncan McVey

USA

Michael McVoy

USA

José A. Melero

Spain

Eyal Meltzer

Israel

Baozhong Meng

Canada

Shanshan Meng

China

Songdong Meng

China

Xiang-Jin Meng

USA

Laura Menotti

Italy

Michael Metzger

USA

Francois Meurens

Canada

Craig Meyers

USA

Luiz Miletti

Brazil

Christopher Miller

USA

Zhen Ming-Hua

China

Zhen Ming-Hua

China

Valerie Mioulet

UK

Chad Mire

USA

Michi Miura

Japan

Tatuso Miyamura

Japan

Susanne Modrow

Germany

Ugo Moens

Norway

Shyam Mohapatra

USA

Sophie Molia

Mali

Debasis Mondal

USA

Peter Monk

UK

Isabella Monne

Italy

M Anthony Moody

USA

Thomas Moran

USA

Luciano Moreira

Brazil

Enrique Moriones

Spain

Igor Morozov

USA

Nuria Morral

USA

Lynda Morrison

USA

Trudy Morrison

USA

Bruno Mota

Brazil

Martin Muggeridge

USA

Patricia Mulrooney-Cousins

Canada

Vincent Munster

USA

Takayuki Murata

Japan

Manoj Murhekar

India

Eain Murphy

USA

Alexander Murray

UK

Jayaseelan Murugaiyan

Germany

Jonah Musa

Nigeria

Joe Mymryk

Canada

Noriyo Nagata

Japan

Venugopal Nair

UK

Yuchen Nan

USA

Krishna Narayanan

USA

Somanna Naveen

USA

Oscar Negrete

USA

Martha Nelson

USA

Rachel Nelson

USA

Lev Nemchinov

USA

Michael Nevels

Germany

Lisa F.P. Ng

Singapore

John Nicholls

Hong Kong

Christian Niel

Brazil

Veljko Nikolin

Germany

Ikuo Nishigaki

Japan

Allan Nix

USA

Richard Njouom

Cameroon

Mauricio Nogueira

Brazil

Elizabeth Norton

USA

Norbert Nowotny

Austria

Akito Nozaki

Japan

Takahiro Ohara

Japan

Seii Ohka

Japan

Kazusato Ohshima

Japan

Tomoichiro Oka

Japan

Margaret Okomo-Adhiambo

USA

Elzbieta Oldak

Poland

Ledy Oliveira

Brazil

Aldemir B. Oliveira-Filho

Brazil

Christopher Olsen

USA

Ken Olson

USA

Tanja Opriessnig

USA

David Ornelles

USA

Hitoshi Oshitani

Japan

Fernando Osorio

USA

Chris Oura

Trinidad and Tobago

Karin Pachler

Austria

Romain Paillot

UK

Betania Paiva Drumond

Brazil

Anandan Paldurai

USA

Minna Paloniemi

Finland

Basu Dev Pandey

Nepal

Glaucia Paranhos-Baccalà

France

Mark Parcells

USA

Leslie Parent

USA

Manmohan Parida

India

Bongkyun Park

Korea, South

Colin Parrish

USA

John Pasick

Canada

Manoj Pastey

USA

Deendayal Patel

USA

Kunj Pathak

USA

Daniela Pavoni

Brazil

Finn Skou Pedersen

Denmark

Niels Pedersen

USA

Ariel Pereda

Argentina

Lenore Pereira

USA

Daniel Perez

USA

Daniel Mariano Perez-Filgueira

Argentina

Martin Pfeffer

Germany

Andreas Pichlmair

Germany

Chamsai Pientong

Thailand

Gorben Pijlman

Netherlands

David Pintel

USA

Aguinaldo Pinto

Brazil

Massimo Pinzani

Italy

Paula Pitha Rowe

USA

Marie Pizzorno

USA

Roman Pogranichniy

USA

Stefan Pöhlmann

Germany

Bernard Poiesz

USA

Stephen Polyak

USA

Malgorzata Pomorska-Mol

Poland

Art Poon

Canada

Leo Poon

Hong Kong

Antonio Postigo

UK

Ali Pouryasin

Iran

Eva Poveda

Spain

Ned Powell

UK

Ramesh Prabhu

USA

Jan Pravsgaard Christensen

Denmark

Krzysztof Pyrc

Poland

Xian Qi

China

Li Qihan

China

Zhuo Ming Qin

China

Hua-Ji Qiu

China

Jorge Quarleri

Argentina

Josep Quer

Spain

Frank Rabenstein

Germany

Sonia Mara Raboni

Brazil

Kathryn Radigan

USA

Marek Radkowski

Poland

Shazia Rafique

Pakistan

Angamuthu Raja

India

Amitis Ramezani

Iran

Jose Manuel Ramos

Spain

Richard Randall

UK

Parmjeet Randhawa

USA

Eric Rassart

Canada

Luca Rastrelli

Italy

Sebastian Rausch

Germany

Jaana Rautava

Finland

Jo Lynne Raymond

USA

Helena Rebelo-de-Andrade

Portugal

Sanjay Reddy

USA

Richard Reeve

UK

Udo Reichl

Germany

Ilona Reimann

Germany

Xiaofeng Ren

China

Julio Reyes-Leyva

Mexico

Robert Ricciardi

USA

Stephen Rice

USA

Juergen Richt

USA

Cornelia Richter

Germany

Julia Ridpath

USA

Guus Rimmelzwaan

Netherlands

Espen Rimstad

Norway

Sally Roberts

UK

Fernando Rodriguez

Spain

David Rodriguez-Lazaro

Spain

Paulo Roehe

Brazil

Camila Romano

Brazil

Rachel Roper

USA

Pierre Roques

France

Rafael Diego Rosa

Brazil

German Rosas-Acosta

USA

Helene Rosenberg

USA

Paul Rota

USA

Adib Rowhani

USA

Raymond Rowland

USA

Brent Ryckman

USA

Wi-Sun Ryu

Korea, South

Evangelista Sagnelli

Italy

Abdel Rahman Said

Egypt

Bruno Sainz

Spain

Renate Sakate

Brazil

Nitin Saksena

Australia

Toshie Sakuma

USA

Jun-ichi Sakuragi

Japan

Siba Samal

USA

Sweety Samal

USA

Rosemary Sang

Kenya

Flavia Santos

Brazil

Ricardo Santos

Portugal

Daniel Santos Mansur

Brazil

Anderson Sa-Nunes

Brazil

Maria Saponari

Italy

Bruno Sargueil

France

Giuseppe Sarli

Italy

Jaya Sastri

USA

Vijaya Satchidanandam

India

Christian Sauder

USA

Bertrand Saunier

France

Barry James Saville

Canada

Hirofumi Sawa

Japan

Luis Schang

Canada

Leonardo Schiavon

Brazil

Horst Schirrmeier

Germany

Mark Schleiss

USA

Scott Schmid

USA

Juergen Schneider-Schaulies

Germany

Bruce Schoneboom

USA

David Schriemer

Canada

Joerg Schuettrumpf

Germany

Matthias Schweizer

Switzerland

Christoph Seeger

USA

Michel Segondy

France

Renata Servan De Almeida

France

Donald Seto

USA

Norazizah Shafee

Malaysia

Shahida Shah

Pakistan

Liang Shang

USA

Shaobin Shang

USA

Yingli Shang

USA

Colin Sharp

UK

Huigang Shen

China

Zhengli Shi

China

Hiroyuki Shimizu

Japan

Kazuya Shirato

Japan

Zabihollah Shoja

Iran

Nathan Shores

USA

Saurabh Shrivastava

India

Maureen Shuh

USA

Deepak Shukla

USA

Matthias Sieber

Germany

Sanna Sillankorva

Portugal

Breno Silva

Brazil

Malgorzata Simm

USA

Thaís Sincero

Brazil

John Sinclair

UK

Priyanka Singh

India

David Skibinski

Singapore

Betty Slagle

USA

Richard David Sloan

UK

Krzysztof Smietanka

Poland

Redmond Smyth

France

Christopher Snyder

USA

Francisco Sobrino

Spain

Maria Söderlund-Venermo

Finland

Jiuzhou Song

USA

Frederic Sorgeloos

Belgium

Ricardo Soto-Rifo

Chile

Daniele Souza

Brazil

Thiago Souza

Brazil

Enea Spada

Italy

Ellen Sparger

USA

Stephen Spatz

USA

Fernando Spilki

Brazil

Cathy Staedel

France

Peter Staeheli

Germany

Robert Stahelin

USA

Karl Stahl

Sweden

John Stambas

Australia

Elspeth Steel

UK

Eike Steinmann

Germany

Drake Stenger

USA

Joanna Stewart

New Zealand

Patricia Hermes Stoco

Brazil

Cristina Stoyanov

USA

Ivan Stratov

Australia

Stephen Stray

USA

André Streck

Brazil

Rebecca Strong

UK

Yvonne Su

Singapore

Mysore Sudarshana

USA

Chris Sullivan

USA

Sheena Sullivan

Australia

Rebecca Sumner

UK

Chaomin Sun

USA

Liying Sun

China

Sujatha Sunil

India

Camille Sureau

France

Manika Suryadevara

USA

Veluvarthy Suryanarayana

India

Gerd Sutter

Germany

Troy Sutton

USA

Fumitaka Suzuki

Japan

Nobuhiro Suzuki

Japan

William Switzer

USA

Gulam Syed

USA

Gilda Tachedjian

Australia

Yasuhiro Takeuchi

UK

Takenori Takizawa

Japan

Wenjie Tan

China

Yasuhito Tanaka

Japan

Yong Tang

China

Koki Taniguchi

Japan

Tawesak Tanwandee

Thailand

Mi-Hua Tao

Taiwan

Xiaorong Tao

China

Zexin Tao

China

Paul Targett-Adams

Sweden

Geraldine Taylor

UK

John Taylor

USA

Jens Peter Teifke

Germany

Bud C. Tennant

USA

Robert Tesh

USA

Robert Thimme

Germany

Paul Thomas

USA

Zhijun Tian

China

Peter Tijssen

Canada

Suresh Tikoo

Canada

Laurence Tiley

UK

Kelvin To

Hong Kong

Stephen Mark Tompkins

USA

Huahua Tong

USA

Noel Tordo

France

Jenny-Ann Toribio

Australia

Montserrat Torremorell

USA

Gloria Trallero

Spain

Alfonsina Trento

Spain

Joanne Trgovcich

USA

Morten Tryland

Norway

David Tscharke

Australia

Chien-Te Kent Tseng

USA

Konstantin Tsetsarkin

USA

Koji Tsujimura

Japan

Ikuo Tsunoda

USA

Tamás Tuboly

Hungary

Tom Tucker

USA

Dann Turner

UK

Stuart Turville

Australia

Mudit Tyagi

USA

Sukathida Ubol

Thailand

Takamasa Ueno

Japan

Susan Uprichard

USA

Thomas Urban

USA

Cica Urbino

France

Astrid Vabret

France

Felipe Vaca

France

Thomas Vahlenkamp

Germany

Steven Van Borm

Belgium

Lia van der Hoek

Netherlands

Tonja van der Kuyl

Netherlands

Wim Van der Poel

Netherlands

Sabine van der Sanden

USA

Erhard van der Vries

Netherlands

Sylvia van Drunen Littel-van den Hurk

Canada

Formijn J van Hemert

Netherlands

Maria Van Kerkhove

UK

Carine Van Lint

Belgium

Ester Vanni

Italy

Surender Vashist

UK

Michael Veit

Germany

Aldo Venuti

Italy

Helene Verheije

Netherlands

Elisa Vicenzi

Italy

Matias Victoria

Uruguay

Carmen Vieira

Brazil

Mauro Viganò

Italy

Dhanasekaran Vijaykrishna

Singapore

Manuel Vilanova

Portugal

Stefan Vilcek

Slovakia

Livia Villar

Brazil

Francois Villinger

USA

Amy Vincent

USA

Sebastian Voigt

Germany

Heiner von Buttlar

Germany

Peter Walker

Australia

Per Wallgren

Sweden

Edward Walsh

USA

Hongquan Wan

USA

Xiu-Feng (Henry) Wan

USA

Chang Yi Wang

USA

Jianhua Wang

China

Jichun Wang

China

Kening Wang

USA

Lihua Wang

USA

Manli Wang

China

Ning Wang

China

Robert YL Wang

Taiwan

Tianyi Wang

USA

Wenbing Wang

China

Xiaofeng Wang

China

Xue Wang

USA

Stacey Ward

USA

Gulam Waris

USA

Matti Waris

Finland

Koichi Watashi

Japan

Roger Watson

UK

Hongping Wei

China

Taiyun Wei

Canada

Manfred Weidmann

Germany

Jason Weinberg

USA

Hana Weingartl

Canada

Herbert Weissenböck

Austria

Sonja Welsch

Netherlands

Kerstin Wernike

Germany

Michelle West

UK

Denise Whitby

USA

Chris Whitehouse

USA

Rebecca Wilkes

USA

Kim Wilson

Australia

Helen Wise

UK

Harald Wodrich

France

Frank Wong

Australia

Kum Thong Wong

Malaysia

Sek Man Wong

Singapore

Sook San Wong

USA

Charles Woods

USA

Stefan Worgall

USA

Pryscilla Wowk

Brazil

Grzegorz Jan Wozniakowski

Poland

Hongzhuan Wu

USA

Ho-sheng Wu

Taiwan

Jaw-Ching Wu

Taiwan

Qingfa Wu

China

Yasong Wu

China

Betty Wu-Hsieh

Taiwan

Harry Xia

USA

Yan Xiang

USA

Gengfu Xiao

China

Shaobo Xiao

China

Yan Xie

China

Youhua Xie

China

Zhixun Xie

China

Huimin Xu

Canada

Jin Xu

China

Kemin Xu

USA

Lvye Xu

China

Wenbo Xu

China

Seiya Yamayoshi

Japan

Tohru Yanase

Japan

Hanchun Yang

China

Jibing Yang

USA

Kai Yang

China

Kui Yang

USA

Zengqi Yang

China

Zhi Min Yang

China

Yoshihiko Yano

Japan

Melvyn Yap

UK

Chau-Ting Yeh

Taiwan

Jonathan Yewdell

USA

Li Yi

China

Yanbin Yin

USA

Sam Yingst

Thailand

Dongwan Yoo

USA

Liang You

USA

Ming-Lung Yu

Taiwan

Xuejie Yu

USA

Xuemei Yu

USA

Xuping Yu

China

Fan Yuchen

China

Kwok Yung Yuen

Hong Kong

Wu Yunfeng

China

Inigo Zabalgogeazcoa

Spain

Mohammad Zafrullah

USA

Hassan Zaraket

USA

Roland Zell

Germany

Cun Zhang

China

Gui-Hong Zhang

China

HM Zhang

China

Xianren Zhang

China

Yan Zhang

China

Deming Zhao

China

Jincun Zhao

USA

Yanlin Zhao

China

Michael Zheng

USA

Ning Zhi

USA

Oleg Zhirnov

Russia

Jin Zhong

China

Bin Zhou

USA

Fengfeng Zhou

China

Guohui Zhou

China

Yi-Hua Zhou

China

Yijun Zhou

China

Guoqiang Zhu

China

Huanzhang Zhu

China

Jiang Zhu

USA

Wu-yang Zhu

China

Xiaoping Zhu

USA

Yiping Zhu

USA

Angelika Ziegler

Germany
